# Anguish and fears about attitude towards Covid-19 vaccines: contrasts between yes and no vax

**DOI:** 10.1007/s44202-022-00038-2

**Published:** 2022-05-12

**Authors:** Alberto Zatti, Nicoletta Riva

**Affiliations:** University of Bergamo, Bergamo, Italy

## Abstract

The present research investigates the possible causes of resistance to vaccination against the COVID-19 virus. A significant percentage of different countries' populations is refractory to being vaccinated (i.e., in October 2021, in Italy, 20% aged 40–50 years old). A 92-item questionnaire was filled in by a sample of 613 subjects, of which 50.4% said they were against COVID-19 vaccines (63.1% female). Guided by the hypothesis that emotionality constitutes a basis of pre-reflective judgment, items relating to fear, anger and anguish were introduced in the survey. The subjects compiled the Difficulties in Emotional Regulation Scale. The differences between the means of the two samples evaluated with the Student test show that it is, above all, the underlying anguish that constitutes the primary discriminant between the two samples. No Vax mainly considers external the sources of anguish, while Yes Vax sources of anguish appear more internal. From this result an interpretation is advanced: it seems more difficult for No Vax to trust authority recommendations/obligations to get vaccinated because anguish is located just outside the one's body, where Authority dominates.

**Supplementary Information:**

The online version contains supplementary material available at 10.1007/s44202-022-00038-2.

## Theoretical introduction

The 2020 pandemic locked down led to months of social distance with several different reactions in the population [6, 23]. Although, if at early 2021, the first vaccines against COVID-19 infection arrived, still in September 2021 a part of the Italian and European population manifests a solid opposition to vaccination. Surveys on the propensity to vaccinate were carried out in different countries [4, 9, 19, 27] reported significant percentages of individuals wary of vaccination. Health authorities of many countries have launched incentive policies for COVID-19 vaccination (see Green Pass in Italy and Europe, but also a lottery, see [25], but failed to involve a relatively large number of people resistant to vaccination. In Italy, as of October 2021, there were still 9,829,232 citizens of the eligible population group who had not been vaccinated against the COVID-19 virus [21].

More recently, some researchers have tried to identify the psychological factors that could explain the anti-vaccine attitude of a part of the No Vax population [5, 32, 34]. Research on correlations with personality traits [26] or forms of intellectualism [20] grasped some psychological variables related to vaccine resistance, although with results that are not entirely evident.

The present study aims to reach a description and interpretation of the attitude toward vaccinating or not concerning the epidemic caused by the COVID-19 virus. The central hypothesis that we intend to test is the following: the bodily experience, that is, the "affective evaluation" that the subjects make of the COVID-19 vaccines, represents the most relevant dimension in the refusal to undergo vaccination. Edelman [10] and Damasio [8] interpret emotionality as the "natural field of the hard core" (so to speak) on which an individual's value system is based (simplified in polarities as: "welcome" vs. rejection; forward vs. away from me; like vs. dislike, etc.).

Mental processes that Kahneman [22] called "fast" would refer to the judgments of pre-reflective apperception [13], in this case, about vaccines. One could explain the rejection of vaccines by saying that vaccination would constitute for those who oppose it, an artificial interference of the biological equilibrium of the person, a technological manipulation by biological sciences on life. Paraphrasing Nietzsche, one could sentence "these vaccines are too human" (emphasis on "too"). It is, therefore, reasonable to think that the apparently irrational attitude of the so-called No Vax rests on a more archaic emotional rationality in which the bodily self opposes its defenses to sub-microscopic attacks, entrenching itself on the principle of inviolability and integrity of one's body.

According to the psychological meaning of Anzieu [2], who focused on the functions carried out by the epidermis as the protection of the integrity of the individual psyche, the anguish of intrusions into one's skin ego could be one of the central psychological factors of the refusal to vaccinate. Ego skin can be intended as the inner experience of the body arising from bodily sensations. It is a significant interface between the psyche and body. Genetically engineered vaccines would be even more feared by those opposed to the vaccine itself, thus because they are characterized by the suspected ability to penetrate even into the cell nucleus [3].

In English, it must be clear that "anguish" is perfectly synonymized with the word "anxiety". Nevertheless, for the theoretical frame of this study, what is intended here with anguish is not a general state of nervousness. Authors formulated a questionnaire that asked about the essential, profound state of "suffering", not referable to anxiogenic events. This could be a reason why in academic social psychology, one can find many articles on the topic of anxiety (for a general review, see books such as [2, 28, 33] but quite a few (very few) work on the topic of social anguish [29, 36]. It is generally assumed that anguish is a valuable concept for clinical phenomena. However, certain historical events can cause the emergence of a mass, although not plenty conscious, anguish mood of a population, like in Soviet Russian XX century domination (see [31]).

Anguish is a psychodynamic construct with an old and profound tradition in philosophy [11]. Anguish is qualified to be a feeling related to an ambiguous object. In the case of health at the pandemic that broke out at the end of 2019, the object in question must undoubtedly refer to the ultimate fate inherent in life itself, namely the possibility of death. Anguish is not just equivalent to a private fear of a referred object. Emotions such as fear and anger mostly have a course that can be defined over time. On the other hand, Anguish appears as a state of the soul, a "place" of the psyche to the depths of which the motivations expressed in the rationalizing conscience are traced back.

Contamination anguish, therefore, multiplies precisely because of the imaginative representation of the "work" of mRNA vaccines. For an example of the fears aroused by mRNA vaccines, see a declaration taken from an Anti-COVID-19 and Anti Green Pass group active on Telegram: "After 6 billion vaccine doses administered, here is what comes out on Science 'It cannot be excluded that the genetic material present in COVID vaccines (DNA in the case of AstraZeneca, Sputnik and J&J; mRNA in the case of Pfizer and Moderna) can be integrated into our genome and can over time exacerbate latent pathologies or create new ones, hence the need for monitoring overtime of this large vaccine experiment.'".

As reported in every social psychology textbook, attitudes are a fundamental construct for interpreting how people react to social events. They can be considered a relatively stable organization of beliefs, feelings, and behavioral tendencies towards objects, groups, events, or socially significant symbols [18]. The psychosocial theory called The Tripartite Model of Attitudes (Affect, Behaviour, Cognition–ABC Model of Attitude–v [1, 7]. constitutes the theoretical background with which authors prepared a new, ad hoc questionnaire. This paper presents the results of the "Affective" side of the attitude.

In one sentence, the central hypothesis of this research is that the No Vax population could be distinguished based on particular anguish, especially the anguish to be invaded into his/her body (following Anzieu–1987–theory of psyche).

## Method

### Participants

A total of 613 subjects from Italy (56.4%) and other European countries (11.7% from Polish, 8.5% from Britain, and a minor percentage from other 15 EU countries, such as Greece 4.2%, France, 2.1%, etc.) undertook this study. 542 participants responded to the entire survey, while 71 did not respond to the sections on anger and anguish. Table 1 shows subjects against vaccination (50.4%) and the declared gender (63.1% female). The sample was collected online via Prolific services.

**Table 1:** Sample descriptives

In favor of the Vaccine	304	49.6
Against the Vaccine	309	50.4
Total	613	100.0
Female	387	63.1
Male	225	36.7
Total	612	99.8
Missing	1	0.2
Partial sample Without item Anger and Anguish	71	11.6
Sample with all items	542	85.5
Total	613	100.0

### Procedure

Data were collected online in September 2021. A survey composed of an ad hoc 92 items (presented in a random order to avoid possible anchoring phenomena) and two additional self-report measures were administered to participants.

### Measures

Data were collected using an original questionnaire, a validated scale on emotional difficulties, and a projective test.

*Ad hoc questionnaire.* An original questionnaire was prepared based on the results of an online (via Telegram) focus group with No Vax declared activists. On a six-value scale, the questionnaire proposed statements concerning which the subject had to choose his/her point of agreement (1 not at all agreed, 6 very much agreed). The questionnaire aimed to collect the subject's opinion on his/her social positioning about vaccination [16]. The complete questionnaire is available at OSF URL https://osf.io/afjsc/?view_only=a93b9e9bccf140c6b1ec910c3d0f2c50.

A series of questions required the level of agreement on claims regarding the obligation to vaccinate and the so-called Green Pass (Opinion Section); how much the subject trust administrative, health, pharmaceutical, and information institutions (Trust Section); questions on individual choices versus public choices (Values Section). These sections aim to probe the Cognitive and Behaviour component of the attitude towards vaccines but will not be analysed here.

Statements about "anger" and "fear" required subjects to indicate, on a scale of 1 (not at all) to 6 (very much), how much they agreed about phenomena inherent pandemic, what it did their fear or anger about vaccines and the presence of the virus in general. Another series of items instead asked how much they assessed the possible circumstances eliciting anguish, not necessarily related to the context of the pandemic in progress. These sections aim to detect the Affective component of the attitudes.

*Emotion dysregulation* The Difficulties in Emotion Regulation Scale (DERS; [15]) is a 36-items self-report measure of emotional difficulties. The DERS is validated in various countries worldwide [14, 24, 37]. It consists of 36 verbal items, which can be organized into six factors. Together with the total sum of the scores, the DERS scale would indicate on which levels of the phenomenology of emotions an individual expresses difficulty in regulating them, such as Awareness and Clear understanding of emotions, Acceptance of them, control of Impulsive behavioral responses, and success, to strive for Goals when turmoiled, and, finally, the use of flexible emotional regulation Strategies.

*Body Image and Schema test (BIST)* The BIST is an original test that authors are developing. It consists of 26 strips made up of four images each, one of which is chosen by the respondent on the basis of "the one that strikes you the most". The BIST is currently on validation. The test is projective in essence because it proposes four symbolic figures for each item without requiring verbal processing. The strips present images that follow a criterion of increasing complexity, from the simplest to the most complex. The 26 items are also organized to give indications of bodily consciousness, according to an ontogenetic criterion: the body schema [32, 38]. The actual version (third edition) has been completed by 8059 subjects collected worldwide by Amazon Mechanical Turk. A factor analysis highlight a structure of eight factors (results in press) listed below with the relative evocative names assigned to them by the research team: (1) heart—the first sign of life—Factor "Proto bodily self"; (2) breath, or the "complementary body self" factor; (3) skin, "Ego skin" factor; (4) genitality, "Ego sex" factor; (5) psychomotor development, "Psychomotor ego" factor; (6) the relational self, "Bonds" Factor; (7) self-image, "Self" factor; (8) the image of the male and female body, "Body Imaginary" factor.

The application of an "in validation process" test like BIST could be judged not so opportune, but, as the results will demonstrate, exploring the affective dimension with a projective scale will result in a meaningful way to describe human emotions. The complete Body Image and Schema Test is visible at URL https://osf.io/27hrm/?view_only=e1f0f52e1e734cfca3ebac4baa36ea60

### Statistical analysis

Data were initially analyzed through simple descriptive, including means, standard deviations, frequencies and percentages. We then tested for the presence of significant differences between those favorable to COVID-19 vaccination (i.e., the Yes Vax) and those opposed to it (i.e., the No Vax) on all items of the survey -as well as on the mean score to the DERS scale and BIST scale- through independent samples t-tests and a p-value ≤ 0.05 was deemed significant. All analyses were performed with SPSS version 26.

## Results

Those favorable toward the COVID-19 vaccine reported a lower anger index than those against vaccinations. The Anger Section of the circumstances introduced by the COVID-19 pandemic, which summarizes the relative items of the questionnaire, presents a t = 5.99, df = 487, p < 0.05 = 95%, Conf. Int [0, 33, 0.66], in the sample against vaccines M = 4.40, SD = 0.88, compared to the sample favourable to vaccines with M = 3.90, SD = 0.94 (see Table 2). The items belonging to this section that was singularly different on the t-test for a higher average of the No Vax sample are presented in Additional files 1, 2. Unsurprisingly, the anger associated with the COVID-19 pandemic finds the Yes Vax a higher average on the item "In the current state of the COVID-19 pandemic how angry it makes me that someone not careful could infect me" (Yes Vax M = 4.21, SD = 1.57; No Vax M = 2.64, SD = 1.52).

**Table 2:** Means, Standard Deviations and results of the independent sample t-tests between Yes and No Vax on statistically significant Affective psychological variables on Anger, Fear and Anguish investigated in this study

Variable	No Vax	Pro Vax	*t-*value	*DF*	*p*-value
	Mean (SD)	Mean (SD)			
Angry towards the COVID-19 virus Index	4.40 (0.88)	3.91 (0.94)	5.99	487	< .001
COVID-19 Fear Index	3.99 (0.87)	3.46 (0.98)	6.48	528	< .001
Anguish Index	3.99 (1.03)	4.23 (1.01)	− 2.56	487	< .001
Specific anguish item: "In general I guess I would feel anguish if I felt like I was being invaded"	4.47 (1.38)	4.15 (1.37)	2.60	487	< .001

*SD* Standard Deviation, *DF* Degrees of Freedom

For the Fear Section (Table 2), the average of the items asking how much the subjects feared vaccination, presents a statistically significant difference between No Vax (generally higher means) against Yes Vax. On the Student's test: t = 6.48, df = 528, p < 0.05 = 95%, Conf. Int [0.36, 0.68], in the sample against vaccines M = 3.98, SD = 0.87, compared to the sample favourable to vaccines M = 3.46, SD = 0.98.

The section that most found a greater number of items with the mean significantly different on the Student's test is the section on anguish: seven items out of ten counts a higher mean in Yes Vax than No Vax. The Anguish Section records a t = − 2.56, df = 487, p < 0.05 = 95%, Conf. Int [− 0.42, − 0.06], in the sample favourable to vaccines M = 4.23, SD = 1.01, compared to the sample against vaccines M = 3.99, SD = 1.03. However, in the item "In general, I guess I would feel anguish if I felt like I was being invaded", the No Vax sample recorded an average higher than the Yes Vax. (No Vax M = 4.47, SD = 1.38; Yes Vax M = 4.15, SD = 1.37).

The between-group differences on DERS subscale and total scores did not reach significance. None of the factors calculated by the DERS were statistically significant on the t-test. This result suggests that the levels of emotional dysregulation of those who were favorable towards the vaccinations were similar to those who were against it. In other words, the two samples, Yes Vax and No Vax do not differ from each other regarding the difficulties in managing emotions.

If a confident tool such as the DERS was unable to say anything about the affective component of the two samples, another tool, perhaps less authoritative, due to an ongoing validation, was able to point out differences between the sample Yes Vax and No Vax. This tool is the Body Image and Schema test (BIST). Table 3 shows the comparison of the means that the t-test gives as statistically significant differences between the Yes Vax sample and the No Vax sample. Please note that for the individual items, the values range from 1 to 4, while for the factors, the values correspond to the regression calculation carried out by the factor analysis, with mean 0 and standard deviation 1.

**Table 3:** Means, Standard Deviations and results of the independent sample t-tests between Yes and No Vax on statistically significant Body Image and Schema Test Factors and Item

Variable	No Vax	Pro Vax	*t-*value	*DF*	*p*-value
	Mean (SD)	Mean (SD)			
"Item Agitation Vital Rhythm"	2.5146 (1.13)	2.7171 (1.07)	− 2.28	611	< .001
"Item skin as Protection Cover of the Ego"	2.9871 (0.99)	2.7763 (1.03)	2.59	611	< .001
"Item Symbol of Female Genitalia Imago Female Sex"	3.1036 (0.87)	2.9474 (0.78)	2.34	611	< .001
"Item Get Up/‘Agentivity’"	2.8188 (1.03)	2.5559 (0.95)	3.28	611	< .001
Factor “Bonds"	0.0973 (0.97)	− 0.0678 (0.95)	2.13	611	< .001
Factor "Psychomotor"	0.2321 (0.99)	0.0671 (0.98)	2.08	611	< .001

*SD* Standard Deviation, *DF* Degrees of Freedom

As detailed in Table 3, the item "Agitation/vital rhythm" records a higher average in the Yes Vax group. Two single items of the Body Image and Schema test result in a statistically higher Student's test mean always in the No Vax sample. They are items "Skin as protection" and "Item Symbol of the Female Genitals/Imago female sex".

The two significant factors that resulted in significantly different t-test are the "Bonds factor" and, above all, the "Psychomotor ego factor", "reinforced" by the item "Getting up/‘Agentivity’". BIST strip examples are presented in Supplementary file.

## Discussion

The research highlights how emotions such as anger and fear discriminate between the two samples for the judgments implicit in these emotions towards situations created by the COVID-19 pandemic. According to the data collected by this research, the Yes and No Vax samples are not different in the management of emotions in general, as shown by the absence of significant differences on the DERS scale. Even the indices and items relating to fear and anger, in general, do not show particularly significant differences. Only on the attribution of the causes of anger, there are differences between the two groups: a tendency towards introversion of anger by the Yes Vax, while in the No Vax, there is a more significant presence of reactions of anger caused by the impositions to do and not do something (see Table 7 in Supplementary file).

These findings would indicate that the two samples, No and Yes Vax are not distinguishable in managing emotions in general. Something more "profound" in affective life has to call onto the psychological scene. This is anguish, which is not a common concept in social psychology, particularly in Anglo-Saxon culture, also because the word "anguish" has a limited use only in philosophy (see [30]). However, it is appropriately on "anguish to be invaded" that the two samples present differences that allow a better understanding of the reasons for the contrast between the two samples, Yes and No Vax.

In fact, in the No Vax sample, we mainly find anguish resulting from feeling invaded. The higher average recorded by the No Vax sample is significant due to the relative anguish of being invaded, very pertinent to the research hypothesis that predicts an unconscious fear in the No Vax population dependent precisely on the intrusion into the personal ego skin. The No Vax group's anguish comes from sources external to the individual, while for the Yes Vax group, the anguish comes from sources internal to the individual. This difference could explain the distinct attitudes of the two groups towards the pressure to get vaccinated; as for the No Vax, everything that comes from "outside oneself" constitutes a potential threat, while for the Yes Vax, just what is proposed by a collective subject external to oneself constitutes the possibility of saving oneself from anguish whose source is internal to the individual. The anguishes of the Yes Vax sample could be grouped into two categories: a category that includes the loss of references, such as falling apart, feeling the earth opening up, feeling that time has stopped; and a category that collects anguishes with social references, such as not receiving esteem, feeling alone and no longer counting for anyone.

The Yes Vax group has only one item whose average is statistically higher on the t-test, the item that signals anguish and agitation. Also, from this, we can understand the profound difference between the active imagery in the two samples: the Yes Vax is restricted in the narrow space dominated by anxious agitation; the No Vax, on the other hand, is enhanced by an imaginary so flourishing as to invest the same reality.

Strips "Item Skin as protection" and "Item Symbol of the Female Genitals/Imago female sex" refer to the theory of the skin ego developed by Anzieu [2], in particular as regards the protective function of the skin itself. A possible interpretation could be that in the No Vax group, the defensive value of the skin is accentuated: a "sophisticated" barrier, as can be deduced from the images, that keeps away from possible intrusions.

An interpretation of the highest average of the No Vax sample of the BIST Factor "Bonding" could concern the sense of community that has been created in individuals who have chosen to resist the social pressures that push toward COVID-19 vaccination. It should also be emphasized that a particular share of the subjects was "recruited" by this survey on social media such as Telegram, where they gather in debates and organization of public positions precisely of the homogeneous groups of No Vax.

Being part of a cohesive group, practically impermeable to external influences, highly reactive, allows the anguish of being invaded, the only item in which the No Vax record an average higher than the Yes Vax, to take on the appearance of a treatable experience [35]. The extroflexion of the anguish of being threatened in one's bodily integrity, assigning it an adversary, an "enemy", thus allows giving a perspective to the anguish itself, a possible way of solution. Where the individual marked by anguish is powerless, the group transforms that anguish into an irresistible force. From the research data, it is possible to deduce that the anguish of death produced by the threat of being invaded would seem to involve the very foundations of the bodily self in the No Vax.

This anguish seems to find a possibility of meaning through an ideological construction, that set of ideas that can be verbally articulated to give shape to it. Social shared in group ideology reinforce by opposing the specular out-group ideology.

The construction of ideological representations and narratives is the work with which the ego gives shape to anguish itself. Ergo, in the schemes of the collective narrative of the Yes Vax and No Vax groups, we will find the ways and means that allow the individual's expulsion-reclamation of the anguish itself.

The Yes Vax narrative is formed by resorting to authors who use the language of scientific rationality, decidedly well-rooted in evidence obtainable from reality data but poor, however, in symbolic evocativeness. The narrative of No Vax is formed by gathering a multiplicity of voices, which find their most favorable breeding ground in social networks. The jumble of voices that rise from this humus creates an iridescent system of phantasmatic symbols powerfully evocative of the bodily self. The multitude of pseudo-scientific and anecdotal details, news from an "alternative" world, receives its clot from the opposition to the voice of the Authority (state, medical-pharmaceutical). The kaleidoscope of the discursive universe of the different and multiple fringes of the No Vax groups enhances the symbolic fantasy with which the individual bodily self tries to control its own fundamental anguish. On the other hand, the discursive universe of subjects in favor of vaccination rests on the only register of scientific and economic rationality. Science in general and medicine, in particular, restrict de facto symbols of the body and its health because their statute is anti-symbolic (literally in the ancient Greek language, the opposite of symbolic is "dia-ballein", which means "divide, break, clash against", from which the word "diabolic") [12, 17].

## Conclusions and future perspectives

The data collected from the survey allow us to elaborate further research hypotheses made public in future articles. The female No Vax sample is closest in the averages to the Indices to the Yes Vax sample. For example, there is a significant difference in the averages between males and females in the No Vax sample on the anguish items, thus indicating that the female No Vax population experiences these times of pandemic with greater apprehension.

According to this preliminary result, female subjects who declared themselves No Vax could constitute a communication bridge with the health authorities. When the No Vax sample is broken down into males and females, the "awareness" factor of the DERS shows that No Vax females have less difficulty than males in being aware of their emotions.

## Supplementary Information

Below is the link to the electronic supplementary material.

Supplementary file 1 (DOCX 636 KB)

Supplementary file 2 (DOCX 44 KB)

## Data Availability

The data that support the findings of this study are available from the corresponding author upon request.

## Questionnaire and code availability

A sample of the questionnaire on which is based the present research and the code that supports the findings of this study is available at URL https://osf.io/afjsc/?view_only=a93b9e9bccf140c6b1ec910c3d0f2c50.

Body Image and Schema Test can is available at URL https://osf.io/27hrm/?view_only=e1f0f52e1e734cfca3ebac4baa36ea60.
