# Supplementary material for: Anguish and fears about attitude towards Covid-19 vaccines: contrasts between yes and no vax
Source: Discov Psychol. 2022 May 12;2(1):26. doi: 10.1007/s44202-022-00038-2 (PMC9098149; doi:10.1007/s44202-022-00038-2)

# Anguish and Fears About Attitude Towards Covid-19 Vaccines: Contrasts between Yes and No Vax

### Figure Body Image and Schema Test

“Psycho-motor Ego” Factor (Figures 1, 2, 3, 4)

Fig. 1 Item Postural Attitude


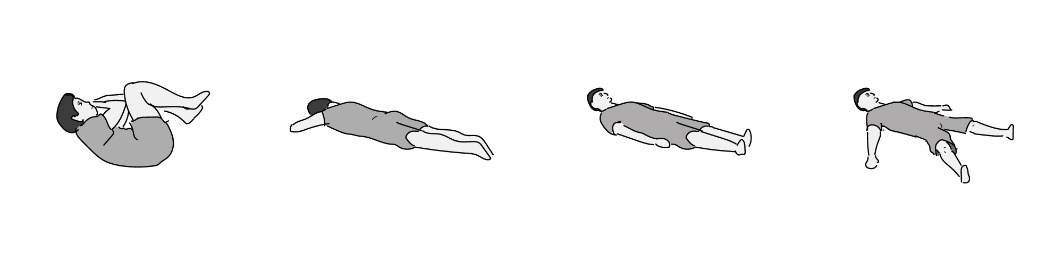


Fig. 2 Item “Agentivity”


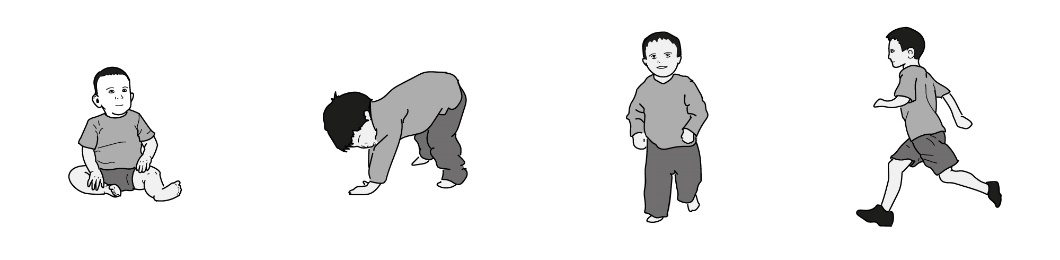


Fig. 3 Item From “Me” to Culture


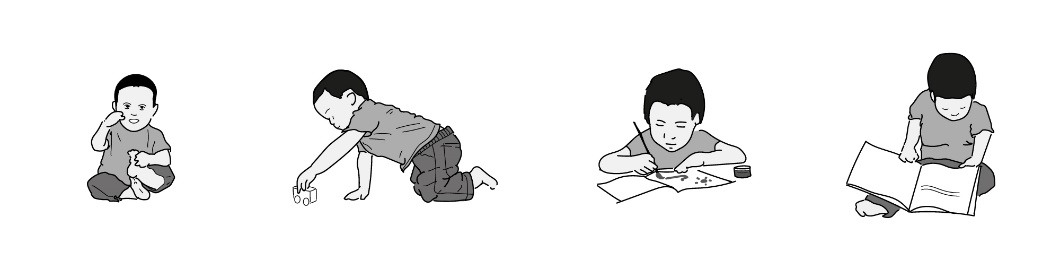


Fig. 4 Item Form Dyad to Group


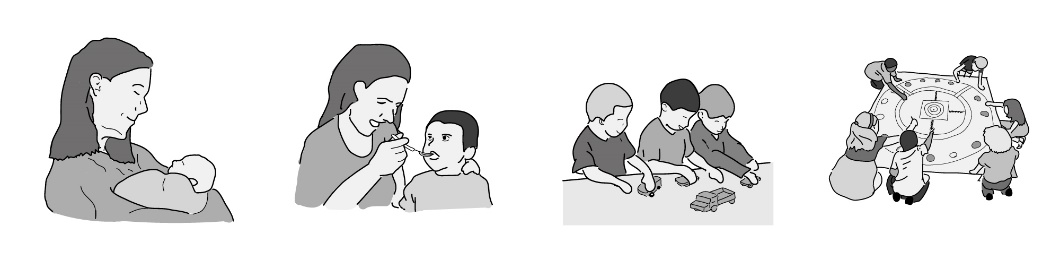


Bonding Factor (Figures 5, 6, 7, 8)

Fig. 5 ItemFusion-Integration


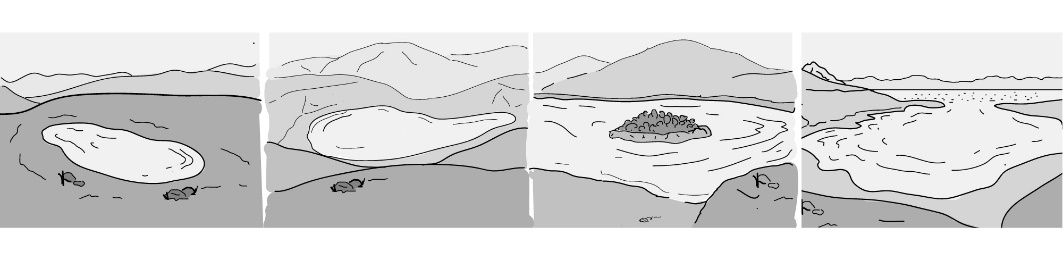


Fig. 6 Item Differentiation-Separation


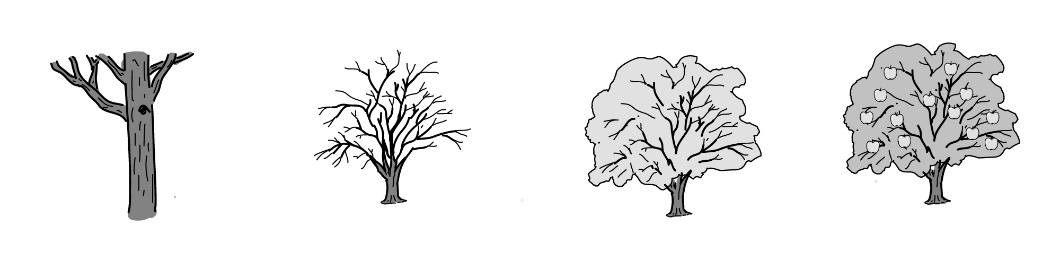


Fig. 7 Item Membership


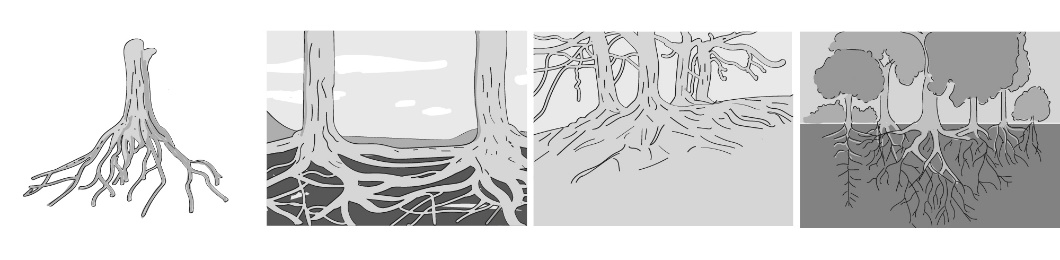


Fig. 8 Item Support


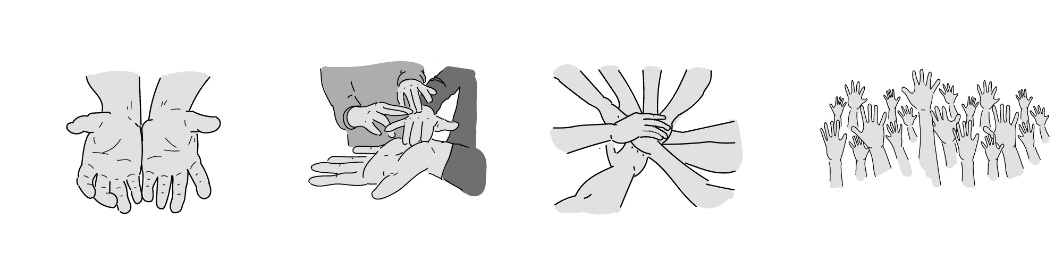


Fig. 9. Item Ego Protection


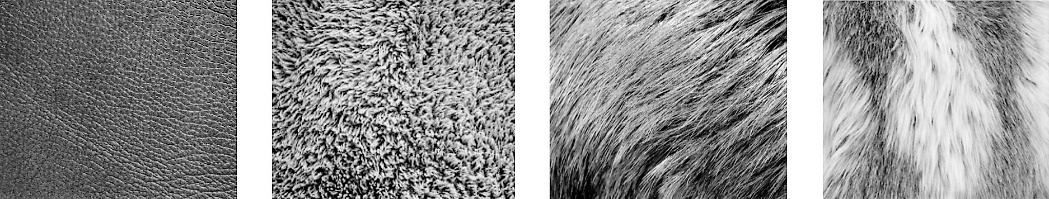


Fig. 10. Item Female Sex Imago


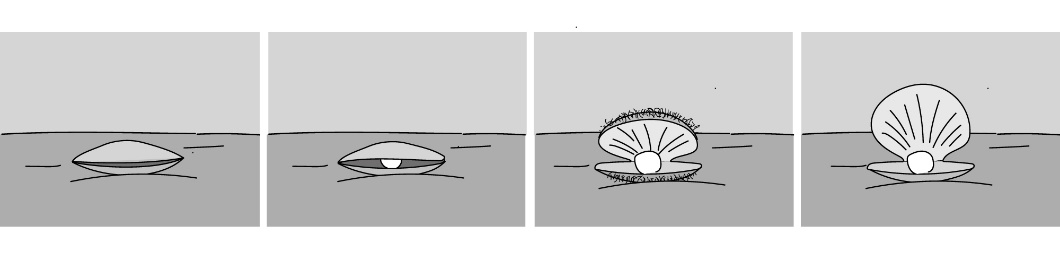


Fig. 11. Item Vital Rhythm


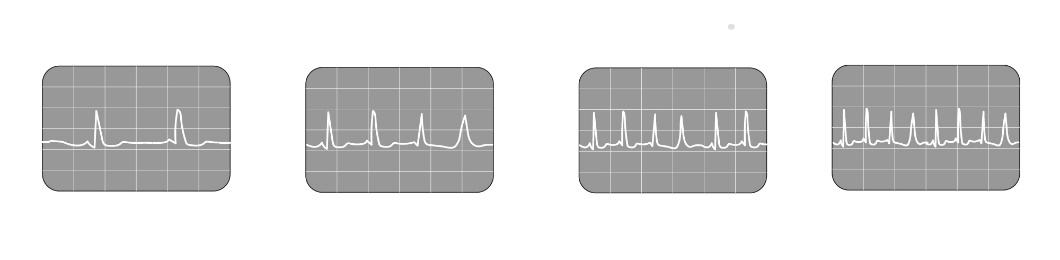

Supplement: Supplementary file 1 — (DOCX 636 KB) [file 44202_2022_38_MOESM1_ESM.docx]
