# Supplementary material for: Anguish and fears about attitude towards Covid-19 vaccines: contrasts between yes and no vax
Source: Discov Psychol. 2022 May 12;2(1):26. doi: 10.1007/s44202-022-00038-2 (PMC9098149; doi:10.1007/s44202-022-00038-2)
Supplement: Supplementary file 2 — (DOCX 44 KB) [file 44202_2022_38_MOESM2_ESM.docx]

## Supplementary Materials

### Tables

Table 4. *Country of residence of all study participants (N = 613)*

|  | Frequency | Percentage |
| --- | --- | --- |
| United Kingdom | 346 | 56.4 |
| Germany | 17 | 2.8 |
| France | 13 | 2.1 |
| Spain | 18 | 2.9 |
| Austria | 2 | .3 |
| Belgium | 3 | .5 |
| Czech Republic | 1 | .2 |
| Denmark | 1 | .2 |
| Finland | 3 | .5 |
| Greece | 26 | 4.2 |
| Hungary | 17 | 2.8 |
| Netherlands | 12 | 2.0 |
| Norway | 2 | .3 |
| Poland | 72 | 11.7 |
| Portugal | 27 | 4.4 |
| Sweden | 1 | .2 |
| United Kingdom | 52 | 8.5 |
| Total | 613 | 100.0 |

Table 5. Between groups *Trust section items different* at *p*-value ≤ .05.

|  | t-test for average equality | | | | |  |  |  |
| --- | --- | --- | --- | --- | --- | --- | --- | --- |
|  | t | Df | Sign. (two-tailed) | 95% difference interval confidence | |  | | |
|  |  |  |  | Inferior | Superior |  | Average | Deviation std. |
| 3) Trust Index | -18.33 | 611 | 0.000 | -1.27 | -1.02 | Vaccine Contrary | 2.17 | 0.71 |
|  |  |  |  |  |  | Vaccine Favorable | 3.32 | 0.84 |
| "Trust in national and local government institutions" | -15.99 | 611 | 0.000 | -1.61 | -1.26 | Vaccine Contrary | 1.80 | 0.98 |
|  |  |  |  |  |  | Vaccine Favorable | 3.23 | 1.22 |
| "Trust in official information" | -15.45 | 611 | 0.000 | -1.54 | -1.19 | Vaccine Contrary | 1.69 | 0.95 |
|  |  |  |  |  |  | Vaccine Favorable | 3.05 | 1.22 |
| "Trust in pharmaceutical companies" | -18.61 | 611 | 0.000 | -1.99 | -1.61 | Vaccine Contrary | 1.70 | 1.03 |
|  |  |  |  |  |  | Vaccine Favorable | 3.50 | 1.35 |
| "Trust in social information – spread from 'below'" | 4.23 | 611 | 0.000 | 0.22 | 0.60 | Vaccine Contrary | 2.72 | 1.35 |
|  |  |  |  |  |  | Vaccine Favorable | 2.32 | 1.02 |
| "Trust in official medicine" | -15.46 | 611 | 0.000 | -1.75 | -1.35 | Vaccine Contrary | 2.96 | 1.36 |
|  |  |  |  |  |  | Vaccine Favorable | 4.51 | 1.11 |

Table 6. Between groups *Position toward pandemic section items different* at *p*-value ≤ .05.

|  | t-test for average equality | | | | |  |  |  |
| --- | --- | --- | --- | --- | --- | --- | --- | --- |
|  | t | Df | Sign. (two-tailed) | 95% difference interval confidence | |  | | |
|  |  |  |  | Inferior | Superior |  | Average | Deviation std. |
| "I have the certainty that my behaviors will not favor the spread of the virus" | 6.15 | 611 | 0.000 | 0.48 | 0.93 | Vaccine Contrary | 4.49 | 1.40 |
|  |  |  |  |  |  | Vaccine Favorable | 3.79 | 1.43 |
| "We must take a clearly contrary position on all the obligations that have been issued" | 14.88 | 611 | 0.000 | 1.43 | 1.87 | Vaccine Contrary | 3.67 | 1.55 |
|  |  |  |  |  |  | Vaccine Favorable | 2.02 | 1.16 |

Table 7. Between groups *Position toward vaccination section items different* at *p*-value ≤ .05.

|  | t-test for average equality | | | | |  |  |  |
| --- | --- | --- | --- | --- | --- | --- | --- | --- |
|  | t | Df | Sign. (two-tailed) | 95% difference interval confidence | |  | | |
|  |  |  |  | Inferior | Superior |  | Average | Deviation std. |
| "How right it is for the state to spend on the pandemic" | -11.12 | 398 | 0.000 | -1.54 | -1.08 | Vaccine Contrary | 1.72 | 1.05 |
|  |  |  |  |  |  | Vaccine Favorable | 3.04 | 1.24 |
| Disadvantages Vs. Advantages of Vaccines" | -12.33 | 398 | 0.000 | -2.04 | -1.48 | Vaccine Contrary | 2.30 | 1.27 |
|  |  |  |  |  |  | Vaccine Favorable | 4.06 | 1.49 |

Table 8. Between groups *Anger section towards Pandemic policy items different* at *p*-value ≤ .05.

|  | t-test for average equality | | | | |  |  |  |
| --- | --- | --- | --- | --- | --- | --- | --- | --- |
|  | t | Df | Sign. (two-tailed) | 95% difference interval confidence | |  | | |
|  |  |  |  | Inferior | Superior |  | Average | Deviation std. |
| COVID-19 Angry Index | 5.99 | 487 | 0.000 | 0.33 | 0.66 | Vaccine Contrary | 4.40 | 0.88 |
|  |  |  |  |  |  | Vaccine Favorable | 3.91 | 0.94 |
| "At the current state of the COVID-19 pandemic how angry it makes me that someone decides on my health" | 13.39 | 487 | 0.000 | 1.46 | 1.96 | Vaccine Contrary | 5.07 | 1.23 |
|  |  |  |  |  |  | Vaccine Favorable | 3.36 | 1.60 |
| "At the current state of the COVID-19 pandemic how much it makes me angry that someone not attentive can infect me" | -11.18 | 487 | 0.000 | -1.85 | -1.30 | Vaccine Contrary | 2.64 | 1.52 |
|  |  |  |  |  |  | Vaccine Favorable | 4.21 | 1.57 |
| "In the current state of the COVID-19 pandemic how angry it makes me that health information can be exploited politically" | 3.42 | 487 | 0.001 | 0.16 | 0.60 | Vaccine Contrary | 5.22 | 1.23 |
|  |  |  |  |  |  | Vaccine Favorable | 4.84 | 1.22 |
| "At the current state of the COVID-19 pandemic how angry it makes me not being able to follow my times in making decisions for my health" | 11.12 | 487 | 0.000 | 1.21 | 1.72 | Vaccine Contrary | 4.68 | 1.39 |
|  |  |  |  |  |  | Vaccine Favorable | 3.22 | 1.51 |

Table 9. Between groups *Anger in general section items different* at *p*-value ≤ .05.

|  | Test t for equality of averages | | | | |  |  |  |
| --- | --- | --- | --- | --- | --- | --- | --- | --- |
|  | t | Df | Sign. (two-tailed) | 95% difference interval confidence | |  | | |
|  |  |  |  | Inferior | Superior |  | Average | Deviation std. |
| "In general. I can say that I get angry when limits are imposed onme" | 4.80 | 487 | 0.00 | 0.36 | 0.87 | Vaccine Contrary | 4.01 | 1.42 |
|  |  |  |  |  |  | Vaccine Favorable | 3.40 | 1.38 |
| "I n general. I can say that I getangry when I'm pushed to do something I don't want" | 5.34 | 487 | 0.00 | 0.43 | 0.93 | Vaccine Contrary | 4.64 | 1.36 |
|  |  |  |  |  |  | Vaccine Favorable | 3.96 | 1.43 |

Table 10. Between groups *Reaction to anger section items different* at *p*-value ≤ .05.

|  | t-test for average equality | | | | |  |  |  |
| --- | --- | --- | --- | --- | --- | --- | --- | --- |
|  | t | Df | Sign. (two-tailed) | 95% difference interval confidence | |  | | |
|  |  |  |  | Inferior | Superior |  | Average | Deviation std. |
| "In general. I can say that when I get angry I get angry I get angry with myself" | -3.05 | 487 | 0.002 | -0.70 | -0.15 | Vaccine Contrary | 2.66 | 1.46 |
|  | -3.01 | 429.089 | 0.003 | -0.70 | -0.15 | Vaccine Favorable | 3.09 | 1.60 |
| "In general. I can say that when I get angry I get angry with anything" | -1.98 | 487 | 0.048 | -0.53 | 0.00 | Vaccine Contrary | 2.33 | 1.42 |
|  | -1.96 | 434.709 | 0.050 | -0.53 | 0.00 | Vaccine Favorable | 2.59 | 1.52 |

Table 11. Between groups *Fear Covid-19 section items different* at *p*-value ≤ .05.

|  | t-test for average equality | | | | |  |  |  |
| --- | --- | --- | --- | --- | --- | --- | --- | --- |
|  | t | Df | Sign. (two-tailed) | 95% difference interval confidence | |  | | |
|  |  |  |  | Inferior | Superior |  | Average | Deviation std. |
| COVID-19 Fear Index | 6.48 | 528 | 0.00 | 0.36 | 0.68 | Vaccine Contrary | 3.99 | 0.87 |
|  |  |  |  |  |  | Vaccine Favorable | 3.46 | 0.98 |
| "Fear that vaccines could harm my health" | 14.45 | 528 | 0.00 | 1.50 | 1.97 | Vaccine Contrary | 5.02 | 1.20 |
|  |  |  |  |  |  | Vaccine Favorable | 3.29 | 1.56 |
| "Fear that someone will make decisions on my skin" | 12.23 | 528 | 0.00 | 1.19 | 1.64 | Vaccine Contrary | 5.02 | 1.22 |
|  |  |  |  |  |  | Vaccine Favorable | 3.61 | 1.44 |
| "Fear that the majority of the population will determine my behavior" | 5.92 | 528 | 0.00 | 0.53 | 1.05 | Vaccine Contrary | 4.30 | 1.58 |
|  |  |  |  |  |  | Vaccine Favorable | 3.51 | 1.46 |
| "Fear of becoming a vehicle of contagion" | -10.02 | 528 | 0.00 | -1.45 | -0.97 | Vaccine Contrary | 2.51 | 1.35 |
|  |  |  |  |  |  | Vaccine Favorable | 3.72 | 1.42 |
| "Fear of becoming a simple number in the statistics of a society" | 6.17 | 528 | 0.00 | 0.62 | 1.19 | Vaccine Contrary | 3.90 | 1.71 |
|  |  |  |  |  |  | Vaccine Favorable | 2.99 | 1.65 |

Table 12. Between groups *Reacion to fear section items different* at *p*-value ≤ .05.

|  | t-test for average equality | | | | |  |  |  |
| --- | --- | --- | --- | --- | --- | --- | --- | --- |
|  | t | Df | Sign. (two-tailed) | 95% difference interval confidence | |  | | |
|  |  |  |  | Inferior | Superior |  | Average | Deviation std. |
| "When I'm afraid. I want to shut myself up" | -2.35 | 467 | 0.02 | -0.64 | -0.06 | Vaccine Contrary | 3.14 | 1.58 |
|  |  |  |  |  |  | Vaccine Favorable | 3.49 | 1.58 |

Table 12. Between groups *Anguish section items different* at *p*-value ≤ .05.

|  | t-test for average equality | | | | |  |  |  |
| --- | --- | --- | --- | --- | --- | --- | --- | --- |
|  | t | Df | Sign. (two-tailed) | 95% difference interval confidence | |  | | |
|  |  |  |  | Inferior | Superior |  | Average | Deviation std. |
| Anguish Index | -2.56 | 487 | 0.01 | -0.42 | -0.06 | Vaccine Contrary | 3.99 | 1.03 |
|  |  |  |  |  |  | Vaccine Favorable | 4.23 | 1.01 |
| "In general. I guess I would feel anguish if I felt myself falling apart" | -2.96 | 487 | 0.00 | -0.62 | -0.12 | Vaccine Contrary | 3.96 | 1.41 |
|  |  |  |  |  |  | Vaccine Favorable | 4.34 | 1.34 |
| "In general. I guess I would feel anguish if I felt the ground open under my feet" | -2.09 | 487 | 0.04 | -0.56 | -0.02 | Vaccine Contrary | 4.22 | 1.55 |
|  |  |  |  |  |  | Vaccine Favorable | 4.50 | 1.43 |
| "In general. I guess I would feel anguish if I felt like I was being invaded" | 2.60 | 487 | 0.01 | 0.08 | 0.58 | Vaccine Contrary | 4.47 | 1.38 |
|  |  |  |  |  |  | Vaccine Favorable | 4.15 | 1.37 |
| "In general. I guess I would feel anguish if I felt I wasn't valued" | -3.86 | 487 | 0.00 | -0.74 | -0.24 | Vaccine Contrary | 3.38 | 1.38 |
|  |  |  |  |  |  | Vaccine Favorable | 3.88 | 1.41 |
| "In general. I guess I would feel anguish if I felt I was totally alone" | -3.07 | 487 | 0.00 | -0.72 | -0.16 | Vaccine Contrary | 3.92 | 1.65 |
|  |  |  |  |  |  | Vaccine Favorable | 4.36 | 1.47 |
| "In general. I guess I would feel anguish if I felt that time doesn't go any further" | -2.13 | 487 | 0.03 | -0.58 | -0.02 | Vaccine Contrary | 3.61 | 1.60 |
|  |  |  |  |  |  | Vaccine Favorable | 3.91 | 1.49 |
| "In general. I guess I would feel anguish if I felt I didn't count for anyone anymore" | -3.21 | 487 | 0.00 | -0.72 | -0.17 | Vaccine Contrary | 3.95 | 1.55 |
|  |  |  |  |  |  | Vaccine Favorable | 4.40 | 1.47 |
